# Excitable dynamics through toxin-induced mRNA cleavage in bacteria

**DOI:** 10.1371/journal.pone.0212288

**Published:** 2019-02-22

**Authors:** Stefan Vet, Alexandra Vandervelde, Lendert Gelens

**Affiliations:** 1 Applied Physics Research Group, Vrije Universiteit Brussel (VUB), Brussels, Belgium; 2 Laboratory of Dynamics in Biological Systems, KU Leuven, Leuven, Belgium; 3 Interuniversity Institute of Bioinformatics in Brussels (IB2), VUB-ULB, Brussels, Belgium; 4 Unité de Chronobiologie théorique, Université Libre de Bruxelles (ULB), Brussels, Belgium; University of Edinburgh, UNITED KINGDOM

## Abstract

Toxin-antitoxin (TA) systems in bacteria and archaea are small genetic elements consisting of the genes coding for an intracellular toxin and an antitoxin that can neutralize this toxin. In various cases, the toxins cleave the mRNA. In this theoretical work we use deterministic and stochastic modeling to explain how toxin-induced cleavage of mRNA in TA systems can lead to excitability, allowing large transient spikes in toxin levels to be triggered. By using a simplified network where secondary complex formation and transcriptional regulation are not included, we show that a two-dimensional, deterministic model captures the origin of such toxin excitations. Moreover, it allows to increase our understanding by examining the dynamics in the phase plane. By systematically comparing the deterministic results with Gillespie simulations we demonstrate that even though the real TA system is intrinsically stochastic, toxin excitations can be accurately described deterministically. A bifurcation analysis of the system shows that the excitable behavior is due to a nearby Hopf bifurcation in the parameter space, where the system becomes oscillatory. The influence of stress is modeled by varying the degradation rate of the antitoxin and the translation rate of the toxin. We find that stress increases the frequency of toxin excitations. The inclusion of secondary complex formation and transcriptional regulation does not fundamentally change the mechanism of toxin excitations. Finally, we show that including growth rate suppression and translational inhibition can lead to longer excitations, and even cause excitations in cases when the system would otherwise be non-excitable. To conclude, the deterministic model used in this work provides a simple and intuitive explanation of toxin excitations in TA systems.

## Introduction

Toxin-antitoxin modules are small genetic elements, omnipresent on the genomes of bacteria and archaea, that code for a small intracellular toxin and its counteracting antitoxin [1–3]. The antitoxin typically has a higher *in vivo* turnover rate than the toxin [4]. In type II toxin-antitoxin modules, both the toxin and the antitoxin are proteins and the toxin neutralization occurs through the formation of non-toxic complexes [5]. In several toxin-antitoxin modules one antitoxin can neutralize up to two toxins, forming either the complex AT or the complex TAT. Toxin-antitoxin modules further have an intricate transcriptional regulation: the antitoxin has a DNA-binding domain with which it can bind to the promoter/operator region of the toxin-antitoxin module, and functions as a weak repressor. The toxin can function as a corepressor or a derepressor for the antitoxin, depending on the toxin:antitoxin ratio [2]. Different toxins have different targets in the cell, for example, CcdB poisons DNA gyrase [6], while MazF and RelE cleave mRNA [7–9]. Such endoribonuclease toxins will be the focus of this paper.

Although toxin-antitoxin modules are widespread in prokaryotes, their biological role is currently still unclear. Toxin-antitoxin modules have been implicated in plasmid maintenance, abortive phage infections, the response of bacterial cells to nutritional stress and the formation of persister cells [3, 10]. These are cells that are tolerant to multiple antibiotics because they are in a temporary state of dormancy [11]. Although previously all known type II mRNA endoribonuclease toxins in *E. coli* K-12 were proposed to be involved in persistence, the role of these toxin-antitoxin modules in persister generation in the absence of stress is currently uncertain [3, 12]. Computational studies can be useful to gain insight into the possible dynamics caused by the architecture of the genetic network and the protein-protein, protein-DNA and protein-RNA interactions in a toxin-antitoxin module. Several groups have studied toxin-antitoxin modules computationally, using either deterministic [13, 14] or stochastic [15, 16] approaches. From these modeling efforts, two possible deterministic explanations have emerged for the elevated free toxin levels that might be linked to the generation of persisters. First, it is plausible that there is bistability between a growing, antitoxin-dominated state and a toxin-dominated state [13, 14, 16, 17]. A critical component to allow the existence of a toxin-dominated state is that higher free toxin levels decrease the cellular growth rates, which in its turn has an effect on the accumulation rate of the toxin itself. Increased noise levels in the presence of stress could lead to stochastic switching between these two states. A second possibility is that the toxin-dominated state only exists as a transient excursion in the free toxin level [15]. Such deterministic excursions could theoretically be generated through a process called excitability, where noise could act to trigger them. Furthermore, if toxins induce growth rate reduction, the duration of such toxin excursions could be significantly lengthened. So far, theoretical studies have only observed such transient toxin excitations using stochastic simulations [15]. As for low molecule numbers the deterministic limit of stochastic models does not always give an accurate description of the real dynamics [18], a potential link to deterministic excitability remains to be shown. Finally, it is important to note that these different types of deterministic dynamics aim at describing the behavior of single cells. Both bistability and excitability can give rise to bimodal distributions on a population level.

In this article we focus on the effect of the cleavage of mRNA in the presence of elevated free toxin levels, which has recently been shown to cause toxin excitations [19]. We use a simplified system, where we leave out the formation of the complex TAT and the transcriptional regulation, as this is the simplest toxin-antitoxin model system that still displays the spikes in the free toxin concentration. We use a deterministic set of differential equations to describe this system and show that it yields similar results as simulations with a Gillespie algorithm. Combining a deterministic approach with a separation of time scales in the system allows to further reduce the problem to a two-dimensional system, which can be visualized and interpreted in the phase plane. Moreover, it facilitates bifurcation analyses that show how changes in system parameters affect the TA dynamics. For example, we verify how nutritional stress, which causes an increase in the degradation rate of antitoxin, influences free toxin spikes. Finally, we examine how additional feedback mechanisms like transcriptional regulation by binding of the toxin-antitoxin complex to DNA, or growth rate modulation and inhibition of translation at high free toxin levels can affect the dynamics of the system.

## Materials and methods

Ordinary differential equations are simulated with the integrate function of the package *scipy* [20] of *python* (Python Software Foundation. Python Language Reference, version 2.7. Available at http://www.python.org). In order to perform bifurcation analysis we use a Newton-Raphson method to solve a set of equations using the jacobian of the system [21].

The stochastic simulations were performed using a Gillespie algorithm, based on treating the biochemical reactions as discrete stochastic events [22], implemented in MATLAB. The concentrations are converted to number of molecules using a volume factor of $3.612\times 10^{8}\;\frac{molecules/cell}{M}$. This factor is based on an estimated volume of an *E. coli* cell of 0.6 (*μm*)^3^ [23] and calculated as $6.02\times 10^{23}\frac{molecules}{mol}\times 1000\frac{l}{m^{3}}\times 6\times 10^{-19}\frac{m^{3}}{cell}$.

A detailed description of the different deterministic and Gillespie models used can be found in the supplementary material (S1 File).

## Results

### Toxin-induced mRNA cleavage leads to toxin excitations

Recently, it has been demonstrated experimentally that the mRNA cleavage by MazF toxins can lead to cell growth heterogeneity [19]. Using stochastic Gillespie modeling, the authors have shown that the growth rate inhibition during stressful conditions may be linked to excitations in the free toxin level, which are only found in simulations if the cleavage of the *mazEF* mRNA itself is included in the model. In this paper, we expand the analysis of this model [19]. Briefly, it describes the transcription of the operon and the translation of the formed mRNA to produce the antitoxin MazE (A) and the toxin MazF (T), taking into account that both proteins form dimers in solution and that the antitoxin is produced at a higher rate than the toxin [4]. The model also includes the dilution of all components due to cell division. Only the mRNA and the antitoxin have a separate, higher degradation rate since these species are less stable than the toxin. The toxin and the antitoxin can bind to form the non-toxic complexes AT or TAT, the latter corresponding to the MazF_2_-MazE_2_-MazF_2_ complex described by Kamada et al. [24]. Since this complex formation has a stabilizing effect on the antitoxin, the degradation rate of the antitoxin in complex is lower than that of the antitoxin alone. Additionally, there is a negative transcriptional regulation, with complex AT binding to the DNA and inhibiting the transcription of the operon. Finally, once the free toxin level exceeds a certain threshold, the toxin will cleave the mRNA of the *mazEF* operon. We made only three adaptations to the model used to describe the *mazEF* toxin-antitoxin system in [19], for the sake of simplicity: when DNA binding is included, only one binding site is considered instead of three; we omit the binding of the antitoxin alone to the DNA, which is known to be much weaker than the binding of toxin-antitoxin complexes [25]; and we exclude the slow unbinding of toxin-antitoxin complexes.

**Fig 1:**
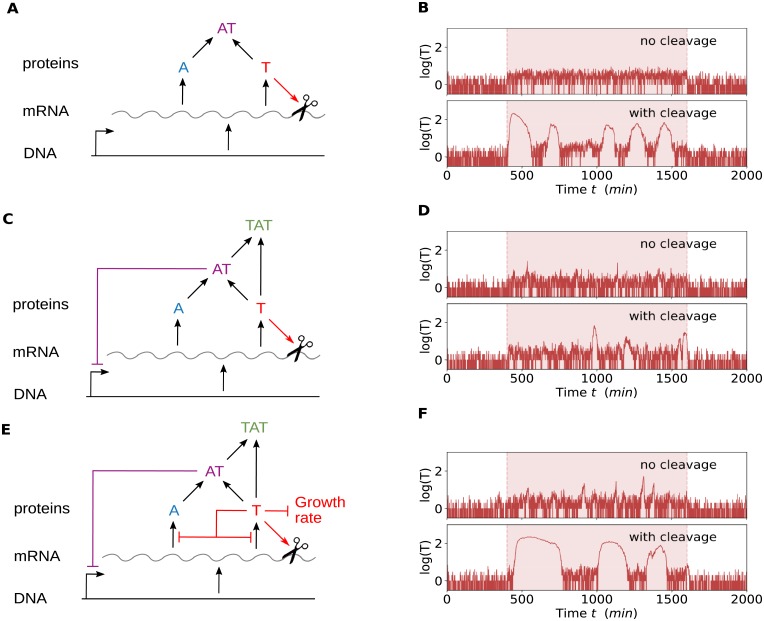
Excitations occur when there is cleavage of mRNA by the toxins. A: Scheme of the minimal system that we use to explain toxin excitations. DNA is transcribed to mRNA which is translated to toxins T and antitoxins A. These can combine to form the complexes AT. If the toxin level reaches a threshold this will cause cleavage of the mRNA. B: Gillespie simulation of the minimal system. In stress conditions (highlighted region) the system is only excitable when mRNA cleavage is included. C: Scheme of the system when including secondary complex (TAT) formation and DNA binding of complex AT, inhibiting the mRNA transcription. D: Gillespie simulation of the system with TAT formation and DNA binding by AT, with and without mRNA cleavage. E: Scheme of the entire system, including translational inhibition as well as inhibition of the growth rate when the toxin level is increased. F: Gillespie simulation of the entire system, with and without mRNA cleavage. Without mRNA cleavage there are increased toxin levels, but pronounced excitory behavior is observed when including mRNA cleavage. For the details of the Gillespie simulations, see supporting information S1 File. Parameters: see Table 1. Additional in (B): *c*_1_ = 1.28 × 10^−5^ *s*^−1^, *c*_2_ = 0.00231*s*^−1^, in (C): *s*_*t*_ = *s*_*m*_ = 1, *b*_*t*_ = *b*_*m*_ = 0.027. In the highlighted regions the stress is increased with a factor two by using *d*_*a*_ = 0.00462*s*^−1^.

In Fig 1 we show the different systems that will be used in this article in order of increasing complexity. The model described above, corresponding to the model used in [19], is shown in the middle panel (Fig 1C). At the top of Fig 1A we show the reduced model, which is our main focus in this article. Finally, for a biologically more complete picture, we analyze the effect of growth rate and translation inhibition at high free toxin levels, as shown in the bottom panel (Fig 1E).

The resulting behavior is shown in Fig 1B, 1D and 1F for the situations with and without mRNA cleavage, where the highlighted zones represent an episode with an increase of stress. As described above, for the model used in [19], we find that, for increased stress, toxin excitations are mainly present if the cleavage of mRNA by the toxins is included in the model (Fig 1D), suggesting that mRNA cleavage plays an essential role in triggering large spikes in the free toxin levels. This made us wonder whether other interactions such as transcriptional regulation by binding of AT to the DNA and the formation of the second complex TAT are also required to generate toxin excitations. When removing these interactions altogether, we found that the qualitative behavior of the system was not affected (Fig 1B). When including mRNA cleavage, similar toxin spikes were observed, while they disappeared when also abolishing the mRNA cleavage. These simulations indicate that toxin-induced mRNA cleavage is one mechanism by which toxin excitations can be triggered. Finally, we wondered whether this behavior persists when adding additional known interactions, such as growth rate modulation and inhibition translation. The growth rate of the cell can be slowed down when toxin levels are increased. This has been included in models before, for example by Cataudella et al. [13]. They show that a combination of growth rate inhibition and conditional cooperativity can lead to a bistable system, with one healthy low-toxin state and one high-toxin state. Additionally, high levels of the MazF toxin can have an influence on the global translation rates in the cell, since MazF also cleaves the transcripts for ribosomal proteins and rRNA precursors [26]. If we include these mechanisms in the Gillespie simulations, we observe toxin excitations, whereby the excitation time is increased due to the growth rate inhibition (Fig 1F), corresponding to the result obtained in [15]. The effect of these mechanisms are analyzed in more detail in the last section.

### Toxin excitations are the result of underlying deterministic excitability

In order to better understand the dynamical origin of the observed spikes in toxin levels (Fig 1), we constructed and analyzed a deterministic model of the reduced TA network, shown in Fig 1C, consisting of ordinary differential equations (ODEs). By not considering the formation of the complex TAT and the transcriptional regulation for now, we focus on analyzing the role of toxin-induced mRNA cleavage in generating toxin excitations. The reduced deterministic model is given by the following 4 ODE equations:

$$\begin{array}{c} \begin{array}{cc} \frac{d[M]}{dt} & =r_{F}-d_{m}\left[M\right]-d_{\text{large}}\frac{{\left[T\right]}^{n}}{{\left[T\right]}^{n}+K_{t}^{n}}\left[M\right],\\ \frac{d[A]}{dt} & =b_{1}\left[M\right]-a_{T}\left[A\right]\left[T\right]-d_{a}\left[A\right],\\ \frac{d[T]}{dt} & =b_{2}\left[M\right]-a_{T}\left[A\right]\left[T\right]-d_{c}\left[T\right]+d_{a2}\left[AT\right],\\ \frac{d[AT]}{dt} & =a_{T}\left[A\right]\left[T\right]-d_{c}\left[AT\right]-d_{a2}\left[AT\right]. \end{array} \end{array}$$ (1)

The parameters and variables can be found in Tables 1 and 2, where most parameters have been experimentally measured or motivated, see [15, 19]. The parameters are identical to those in [19] unless specified below. DNA is transcribed to mRNA [M] with a constant transcription rate *r*_*F*_, 0.121 *s*^−1^, based on a transcription rate of 70 nucleotides per second [27] and the length of the transcript. This mRNA is then translated to antitoxins and toxins with rates *b*_1_, 0.122 *s*^−1^, and *b*_2_, 0.009 *s*^−1^. The toxin translation is lower than the antitoxin translation, this difference was measured experimentally for several type II toxin-antitoxin modules including *mazEF* using ribosome profiling [28]. A toxin and an antitoxin can bind to form a complex AT with a rate *a*_*T*_, 1.32 × 10^5^ *M*^−1^ *s*^−1^, corresponding to 0.000365 *s*^−1^ after applying the volume factor. This value is based on binding experiments using Surface Plasmon Resonance (SPR) (personal communication) and lies within the expected theoretical range [29, 30]. The mRNA has a degradation rate *d*_*m*_ of 0.002 *s*^−1^ under normal conditions (in the absence of stress conditions), based on a half-life of 5.7 minutes [31]. If the toxin level reaches a threshold, then it is assumed that mRNA cleavage is activated, which is modeled by an increase of the degradation rate (*d*_large_) and by using a Hill-function with threshold *K*_*t*_ and coefficient *n*. Here, we assume that the maximal degradation of the mRNA in the presence of the toxin is 100 times larger than *d*_*m*_ (the basal degradation rate of mRNA), while it was fixed at 10 molecules *s*^−1^ in [19]. The Hill coefficient on the other hand is fixed at 2 for all simulations in this paper unless specified differently, while it was 3 in [19]. The threshold *K*_*t*_ is fixed at 15 toxins per cell, as in [19]. The toxin and complex AT are considered as stable and are diluted at a rate *d*_*c*_ of 0.00028 *s*^−1^ as the result of cell division, with an estimated cell cycle length of 40 minutes. The antitoxin is degraded with a higher rate *d*_*a*_, which was estimated as 8 times *d*_*c*_. Although the antitoxin is more stable within toxin-antitoxin complexes, we assume that it can still be degraded in this case with a lower rate *d*_*a*2_. As this depends on the degradation rate of the antitoxin, we assume that the ratio $\frac{d_{a2}}{d_{a}}=0.1$ is fixed. Besides the difference in the degradation rates of the toxin and the antitoxin, the toxin translation rate is smaller as well, introducing a difference in the time scales of A and T. In order to quantify this, we use $\varepsilon =\frac{b_{2}}{b_{1}}⪡1$, and reformulate (1) as follows:

$$\begin{array}{c} \begin{array}{cc} \frac{dm}{d\tau } & =(\frac{1}{\varepsilon }-m-\left(\beta -1\right)\frac{x^{n}}{x^{n}+\kappa^{n}}m),\\ \frac{da}{d\tau } & =-\alpha ax+\gamma m-\delta_{a}a,\\ \frac{dx}{d\tau } & =-\alpha ax+\varepsilon (\gamma m+\delta_{AT}y-\delta_{c}x),\\ \frac{dy}{d\tau } & =\alpha ax-\varepsilon (\delta_{c}+\delta_{AT})y. \end{array} \end{array}$$ (2)

**Table 1:** Redefinition of the parameters for the normalized model and the correspondence to the original parameters. These parameters are valid for non-stress conditions. In case of stress, *δ*_*a*_, *d*_*a*_ and/or *ε* are adjusted as indicated in the figure captions.

Normalized model			Original model		
Parameter	Definition	Value	Parameter	Description	Value
*δ*_*c*_	$\frac{d_{c}}{d_{m}\varepsilon }$	1.96	*d*_*c*_	Decay rate due to cell division	0.00028 *s*^−1^
*δ*_*a*_	$\frac{d_{a}}{d_{m}}$	1.16	*d*_*a*_	Decay rate of antitoxin	0.00231 *s*^−1^
*δ*_*AT*_	$\text{F}\times \frac{d_{a}}{d_{m}\varepsilon }$	$\text{F}\times \frac{\delta_{a}}{\varepsilon }$	*d*_*a*2_	Decay rate of antitoxin within complex AT	F × *d*_*a*_
			*F*	Decrease in antitoxin degradation within complex AT	0.1
			*d*_*m*_	Decay rate of mRNA	0.002 *s*^−1^
*β*	$\frac{d_{\text{large}}}{d_{m}}+1$	100	*d*_large_	Increased mRNA degradation	0.198 *s*^−1^
*α*	$\varepsilon \frac{a_{T}}{d_{m}^{2}}$	6.74	*a*_*T*_	Binding of toxin and antitoxin	0.000365 *s*^−1^
			*r*_*F*_	mRNA transcription rate	0.121*s*^−1^
*γ*	$\frac{r_{F}D}{d_{m}}b_{1}$	7.38	*b*_1_	Antitoxin translation rate	0.122 *s*^−1^
			*b*_2_	Toxin translation rate	0.009 *s*^−1^
*ε*	$\frac{b_{2}}{b_{1}}$	0.0738	*ε*	Ratio of translation rates	0.0738
*κ*	$\frac{d_{m}K_{t}}{\varepsilon }$	0.407	*K*_*t*_	Threshold for cleavage of mRNA	15
*n*		2	*n*	Hill factor	2
			*v*	Volume of an *E. coli* cell	0.6 (*μm*)^3^

**Table 2:** Normalized variables and their correspondence to the real variables.

Normalized model		Original model	
Variable	Definition	Variable	Description
*m*	$\frac{d_{m}}{r_{F}\varepsilon }$ [M]	[M]	mRNA
*a*	$\frac{d_{m}}{\varepsilon }$ [A]	[A]	Antitoxin
*x*	$\frac{d_{m}}{\varepsilon }$ [T]	[T]	Toxin
*y*	$\frac{d_{m}}{\varepsilon }$ [AT]	[AT]	Complex AT
*z*	$\frac{d_{m}}{\varepsilon }$ [TAT]	[TAT]	Complex TAT
*τ*	*d*_*m*_t	t	Time

This model is now normalized in a way that the parameters are approximately of order *O*(1), except for *β* and *ε*. Here, $\beta =(\frac{d_{\text{large}}}{d_{m}}+1)$ quantifies the difference between the minimal and maximal mRNA cleavage rate, and $\varepsilon =\frac{b_{2}}{b_{1}}$ quantifies the difference in the translation rates of the toxin and the antitoxin. The definitions of the new parameters and variables are listed in Tables 1 and 2. As *ε* is small, three different time scales can be distinguished: of order *O*(*ε*) (slow dynamics), order *O*(1) (fast) and order $O(\frac{1}{\varepsilon })$ (very fast). The equations for mRNA (*m*) and antitoxin (*a*) are determined by terms of order *O*(1) or order $O(\frac{1}{\varepsilon })$ and will approach their equilibrium condition relatively quickly in comparison to the toxin (*x*) and the complex AT (*y*) (*O*(*ε*)). As a result, we assume that $\frac{dm}{d\tau }=0$ and $\frac{da}{d\tau }=0$ in order to describe the long term dynamics. This is often called a quasi steady state approximation. Carrying out this further simplification the system becomes:

$$\begin{array}{c} \begin{array}{cc} \frac{dx}{d\tau } & =-\alpha a\left(x\right)x+\varepsilon (\gamma m\left(x\right)+\delta_{AT}y-\delta_{c}x),\\ \frac{dy}{d\tau } & =\alpha a\left(x\right)x-\varepsilon \left(\delta_{c}+\delta_{AT}\right)y, \end{array} \end{array}$$ (3)

with

$$\begin{array}{c} \begin{array}{cc} m(x) & =\frac{1}{\varepsilon (1+\left(\beta -1\right)\frac{x^{n}}{x^{n}+\kappa^{n}})},\\ a(x) & =\frac{\gamma m(x)}{\alpha x+\delta_{a}}. \end{array} \end{array}$$ (4)

**Fig 2:**
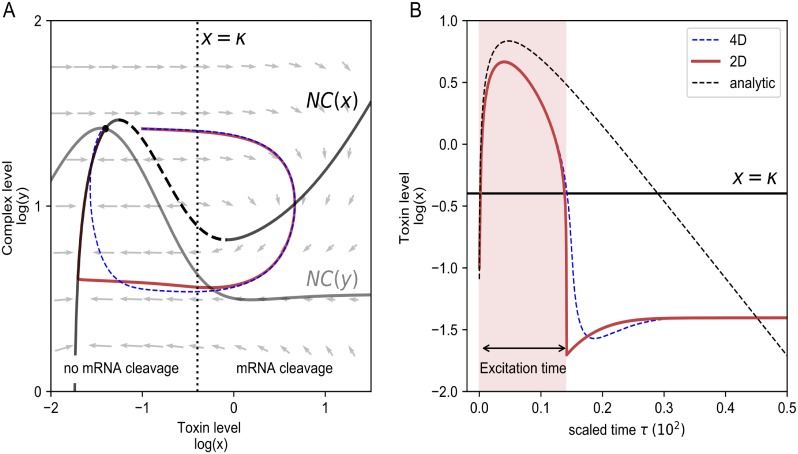
Phase plane visualization (A) and time series (B) of a toxin excitation. A: Phase plane visualization of the simplified 2D system (3) (red) and the 4D ODE system (2) (blue, dashed). The vectors represent the direction of the flow in each point. NC(x) and NC(y) correspond to the nullclines of the system (2), whereby the excitability threshold is marked by the dashed line. The equilibrium is determined by the cross-section of the nullclines. For toxin levels higher than *x* = *κ* the mRNA cleavage is switched on. B: The same trajectories are shown as a time course, together with the analytic estimate (6) of the excitation. We define the excitation time as the time that the toxin level is higher than the threshold for the mRNA cleavage, *κ*. (*δ*_*a*_ = 1.5, *ε* = 0.074.).

Since this reduced system of Eq (3) is two-dimensional, the time evolution of the toxin *x* and complex *y* can be depicted in a simple two-dimensional graph (see Fig 2A). For every set of initial conditions (*x* = *x*_0_, *y* = *y*_0_) in the plane, the Eq (3) predict how the system will evolve. In Fig 2, these predictions are illustrated by the gray arrows, which represent the so-called “flow” of the system. The arrows not only show the direction in which the system evolves, but also the speed with which it does so (larger arrows represent faster dynamics). Such a two-dimensional flow allows us to immediately see by eye how the system will evolve in each situation. Note that the scale used in this figure is logarithmic. Another useful tool to understand the behavior of two-dimensional systems is to plot the nullclines. Nullclines consist of all points (*x*, *y*) where $\frac{dx}{d\tau }=0$ (*NC*(*x*), black line) or $\frac{dy}{d\tau }=0$ (*NC*(*y*), gray line), as given by:

$$\begin{array}{c} \begin{array}{cc} NC(x) & =\frac{\alpha a\left(x\right)x+\varepsilon \delta_{c}x-\varepsilon \gamma m\left(x\right)}{\varepsilon \delta_{AT}},\\ NC(y) & =\frac{\alpha a(x)x}{\varepsilon (\delta_{c}+\delta_{AT})}. \end{array} \end{array}$$ (5)

The intersection of both nullclines represents the equilibrium situation of the system, which is called a fixed point. In our case, there is only one such fixed point and it is stable in the sense that small perturbations will immediately return to this equilibrium. In fact, as it is the only fixed point, all initial conditions (all points in the plane) will eventually return to this equilibrium state. However, when perturbing the system such that the dashed part of NC(x) is crossed, the system will make a large excursion in the plane before returning to the fixed point (see red line in Fig 2A). The excursion in the plane corresponds to a spike in the toxin levels, see Fig 2B. This behavior is called *excitability* [32].

Strictly speaking this analysis in the plane only applies to the reduced two-dimensional system (3). However, we also evaluated the full four dimensional system (2) and projected its dynamics onto the plane (*x*, *y*) (see blue line in Fig 2A). The full model behaves very much alike, exhibiting similar excitable dynamics. This confirms the validity of the quasi steady state approximation used in deriving the reduced model system. Small differences are observed for low toxin levels, in the regime *x* < *κ*, because the time scale separation used in our reduction no longer applies. In this regime, a different normalization could be used, see supporting information, S2 File.

What happens biologically is that when crossing the threshold for mRNA cleavage (*x* > *κ*), mRNA cleavage quickly reduces the levels of mRNA (*m*), which in turn suppresses the translation of antitoxin (*a*) and toxin (*x*), thereby preventing the formation of complexes (*y*). Even though toxin translation is suppressed, at first toxin levels keep increasing as the complexes fall apart with a rate *δ*_*AT*_. When there is an insufficient amount of complexes left, the dilution rate *δ*_*c*_ will dominate so that the toxin level decreases. When the level of toxins drops below the mRNA cleavage threshold again, the toxin excitation is terminated, and the system relaxes to the fixed point with low levels of toxins. These rates *δ*_*c*_ and *δ*_*AT*_ determine the shape of the toxin excitation (Fig 2B) as can also be seen analytically by simplifying the Eq (3) in the limit *x* > > *κ* as follows (for details see supporting information, S2 File):

$$\begin{array}{c} \begin{array}{cc} & x\left(\tau \right)=\left(x\left(0\right)+y\left(0\right)\right)e^{-\varepsilon \delta_{c}\tau }-y\left(0\right)e^{-\varepsilon (\delta_{c}+\delta_{AT})\tau }. \end{array} \end{array}$$ (6)

**Fig 3:**
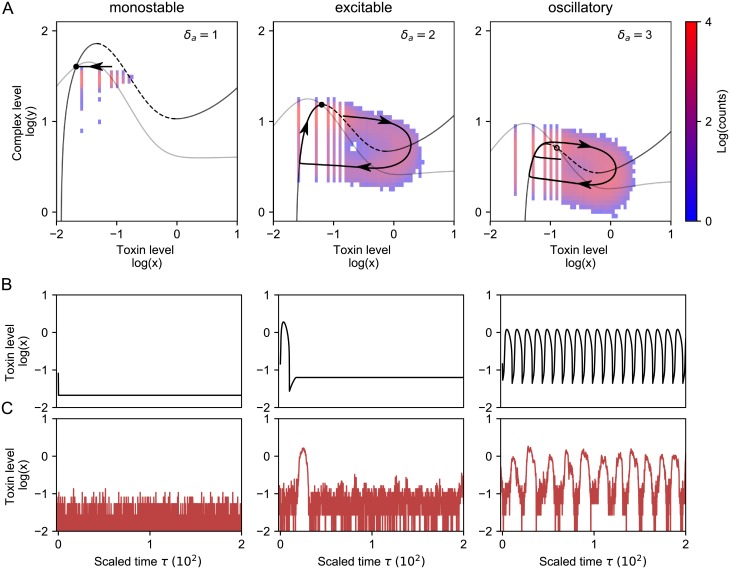
Phase planes (A) and time series of the 2D system (3) (B) and the Gillespie model (C) for monostable, excitable and oscillatory behavior. A: Phase plane representation for monostable (*δ*_*a*_ = 1), excitable (*δ*_*a*_ = 2) and oscillatory (*δ*_*a*_ = 3) behavior. The heatmap corresponds with the amount of times that the Gillespie simulation passes through a local region in phase space. The black trajectory corresponds with the simulation with Eq (3) and follows the path of highest probability obtained from the Gillespie data. Note that values corresponding with toxin levels equal to zero are not shown, due to the logarithmic scale. B: Time traces for the two-dimensional system (3), corresponding to the trajectory in the phase plane. C: Gillespie simulations. The toxin excitations have similar shape as when simulated with the deterministic models.

While our findings show that toxin excitations in a deterministic system are related to excitability, it does not yet prove that the stochastic toxin spikes we observed in Fig 1 and in [19] were related to underlying deterministic excitability. Indeed, various works have shown that excitations can also be a purely stochastic phenomenon without the need for any corresponding deterministic oscillatory behavior [33–35]. In order to test this, we performed simulations with a Gillespie model, using the parameter values corresponding to the ODE model in Fig 2, for different values of the antitoxin degradation rate *δ*_*a*_. We then visualized the data obtained from the stochastic simulation as a heatmap in the plane (*x*, *y*), thus plotting the probability to find the system at a certain location in this two-dimensional space. The results are shown in Fig 3A, where the heatmap illustrates the most probable trajectory of the toxin excitation. This most probable path in phase space nicely overlaps with our deterministic predictions. The corresponding time traces of the deterministic and the Gillespie simulations are represented in Fig 3B and 3C. Together, these simulations confirm that the toxin pulses observed in Gillespie simulations correspond to stochastically triggered pulses, existing due to underlying deterministic excitability.

### The toxin-antitoxin system can be monostable, excitable or oscillatory

Next, we explored the robustness of these toxin excitations to changes in the system parameters. In Fig 3, we show that the system can display qualitatively different behavior when changing the antitoxin degradation rate (*δ*_*a*_). Note that *δ*_*AT*_ varies along with *δ*_*a*_, as the ratio $\frac{\delta_{AT}}{\delta_{a}}$ is fixed. For *δ*_*a*_ = 1, the system has one stable solution and stochastic Gillespie simulations do not show any toxin excitations (Fig 3A). This is explained by the fact that the excitation threshold is too large, such that noisy excursions around the stable state are too small to trigger any excitation. We note that the projected Gillespie data does seem to occasionally cross the threshold (dashed line), even though this does not lead to an excitation. This discrepancy between the Gillespie simulations and the reduced 2D ODE system is explained by the fact that perturbed values of *m* and *a* in the Gillespie simulation do not immediately reach their quasi steady state condition. Whereas an excess of toxins immediately leads to mRNA cleavage in the reduced deterministic case, there is a small delay in the Gillespie simulations. As long as the antitoxin level is sufficiently large, complex formation is able to reduce the toxin level, thus having a stabilizing effect on the system.

When increasing *δ*_*a*_, the excitability threshold is reduced, such that the probability to stochastically excite toxin spikes increases. Indeed, for *δ*_*a*_ = 2, toxin excitations can be observed in the Gillespie simulations (Fig 3B). Although the deterministic 2D model accurately predicts the shape of the toxin excitations, the equilibrium state is often not fully reached in the Gillespie simulations. The reason is that the fixed point is situated close to the top of the nullclines, causing lower complex levels when a perturbation crosses *NC*(*y*), or an excitation when *NC*(*x*) is crossed. When the initial condition is such that it corresponds with a point that is situated in the main band of the Gillespie data, then the deterministic trajectory of the first excitation does correspond well with the Gillespie simulations. This discrepancy disappears when the fixed point is situated further from the top of the nullclines (see also supporting information, S2 Fig).

Finally, for even larger antitoxin degradation rates (e.g. *δ*_*a*_ = 3), the reduced ODE system shows oscillatory behavior (Fig 3C). The fixed point becomes unstable and instead the system converges to a limit cycle. Consistent with these deterministic findings, Gillespie simulations also show more regular excitations.

### Cellular stress causes more excitations

When a bacterial cell is experiencing nutritional stress such as amino acid starvation, the degradation rate of several antitoxins (*δ*_*a*_) increases due to the increased activity of cellular proteases such as Lon [7, 36]. As shown before in Fig 3, this increases the probability of toxin excitations. Another way to influence the toxin level is by directly increasing the translation rate of the toxin (by varying *ε*). Here, using time evolution simulations and bifurcation analysis, we analyze how changes in the system parameters *δ*_*a*_ and *ε* affect the toxin-antitoxin dynamics. Other important biological parameters that affect the dynamics are the parameters determining the mRNA cleavage (*β*, *n*, *κ*) and the binding parameter *α*. The influence of these parameters on the dynamics and shape of the nullclines are discussed in S1 Fig.

**Fig 4:**
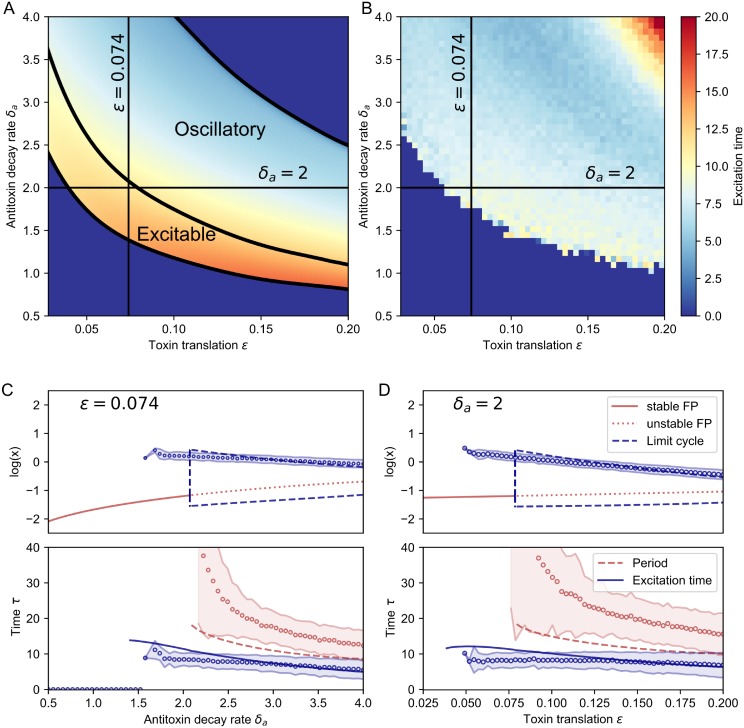
Influence of the antitoxin decay rate *δ*_*a*_ and the toxin translation rate *ε* on the excitation time. A: Heatmap of the excitation time as a function of the antitoxin decay rate *δ*_*a*_ and the toxin translation rate *ε*, using Eq (3). B: Heatmap of the excitation time as a function of the antitoxin decay rate *δ*_*a*_ and the toxin translation rate *ε*, using Gillespie simulations. C: Bifurcation diagram for the antitoxin decay rate *δ*_*a*_, keeping *ε* = 0.074 constant. The system becomes oscillatory in a supercritical Hopf bifurcation. The amplitude of a toxin excitation, the period and the excitation time are compared in the deterministic (solid, dashed and dotted lines) and the Gillespie model (mean: open circles, shaded region: mean ± one standard deviation). D: Bifurcation diagram for the toxin translation rate *ε*, keeping *δ*_*a*_ = 2 constant. Notation same as in C.

In order to analyze the results of the bifurcation analysis (Fig 4) we define the excitation time as the time that the toxin level is higher than the toxin threshold *κ* used in the Hill function (see Fig 2B). Fig 4A and 4B show the average excitation time of toxin excitations, obtained with deterministic (A) and stochastic Gillespie (B) modeling, as a function of the antitoxin decay rate *δ*_*a*_ and toxin translation rate *ε*. Our simulations show that excitations occur for moderate stress levels, while higher stress leads to oscillatory behavior.

When stress levels are increased even further, the fixed point becomes stable again, although we are currently in a state where free toxin dominates the behavior of the system, and this situation would not be viable for a cell. The loss of oscillations is less clear in the Gillespie data, as some excitations are still detected. This is explained by the fact that the toxin value *x* at the fixed point lies close to threshold *κ*, such that noise can still trigger excursions.

Interestingly, in the Gillespie simulations the excitation time remains constant over a large range of *ε* and *δ*_*a*_, whereas in the deterministic case the excitation time is larger for lower values of *δ*_*a*_ and *ε*. The reason is that here the fixed point is situated closer to the top of the nullclines, so that the amplitude of an excitation is slightly larger than in the Gillespie simulation.

This is analyzed in more detail by keeping *ε* = 0.074 fixed in Fig 4C and by keeping *δ*_*a*_ = 2 fixed in Fig 4D. The bifurcation analysis, using Eq (3), shows that the fixed point loses its stability in a supercritical Hopf bifurcation. We found that the amplitude of the limit cycle is increased almost instantaneously in what is called a Canard explosion [37]. The excitability of this system is related to the vicinity of a limit cycle in parameter space. As the period is non-diverging at the bifurcation point, this is an example of type II excitability [38]. The maximum toxin level during an oscillation, the excitation time and the period of an oscillation are compared with the observation in the Gillespie data. They show a good correspondence, although the period of the Gillespie data is larger, as it takes time for a perturbation to cross the excitation threshold. In Fig 4C and 4D we clearly see that the period decreases for increased stress, so that excitations are more frequent. We see a decrease in the excitation time in the deterministic model, while in the Gillespie data the excitation time is more constant. This discrepancy is due to the fact that the Gillespie data does not fully reach the fixed point, as explained in last section. This behavior is illustrated in more detail in S2 Fig, where we explore the system dynamics for different values of *ε*.

### Toxin excitations persist when including additional complex formation and DNA binding

So far, we showed that toxin-induced mRNA cleavage is the main mechanism leading to excitable behavior. Here, we will incorporate the effect of a second complex TAT and the binding of AT to the DNA and show that this does not change the dynamics in a qualitative manner (Fig 1). Each deterministic and corresponding stochastic model is described in more detail in S1 File.

#### Inclusion of a secondary complex

The system (3) can be extended to incorporate the second complex TAT by using an additional variable $z=\frac{d_{m}\left[TAT\right]}{\varepsilon }$. We assume that *y* and *z* have the same creation rate *α*. We also assume that *z* is reduced with rate *δ*_*AT*_ to two toxins *x* due to antitoxin degradation within toxin-antitoxin complexes. The difference in time scales still exists, so that *m* and *a* can be assumed to be in steady state, and the resulting normalized equations are the following:

$$\begin{array}{c} \begin{array}{cc} & \frac{dx}{d\tau }=-\alpha \left(a\left(x\right)+y\right)x+\varepsilon (\gamma m\left(x\right)+\delta_{AT}\left(y+2z\right)-\delta_{c}x),\\ & \frac{dy}{d\tau }=\alpha \left(a\left(x\right)-y\right)x-\varepsilon \left(\delta_{c}+\delta_{AT}\right)y,\\ & \frac{dz}{d\tau }=\alpha xy-\varepsilon \left(\delta_{c}+\delta_{AT}\right)z. \end{array} \end{array}$$ (7)

To show the similarity of this three-dimensional ODE model with the two-dimensional model (3), we look at the total amount of toxin in the complexes, *c* = *y* + 2*z* rather than the dynamics of *y* and *z* separately, resulting in the following system:

$$\begin{array}{c} \begin{array}{cc} & \frac{dx}{d\tau }=-\alpha \left(a\left(x\right)+y\right)x+\varepsilon (\gamma m\left(x\right)+\delta_{AT}c-\delta_{c}x),\\ & \frac{dc}{d\tau }=\alpha \left(a\left(x\right)+y\right)x-\varepsilon \left(\delta_{c}+\delta_{AT}\right)c. \end{array} \end{array}$$ (8)

**Fig 5:**
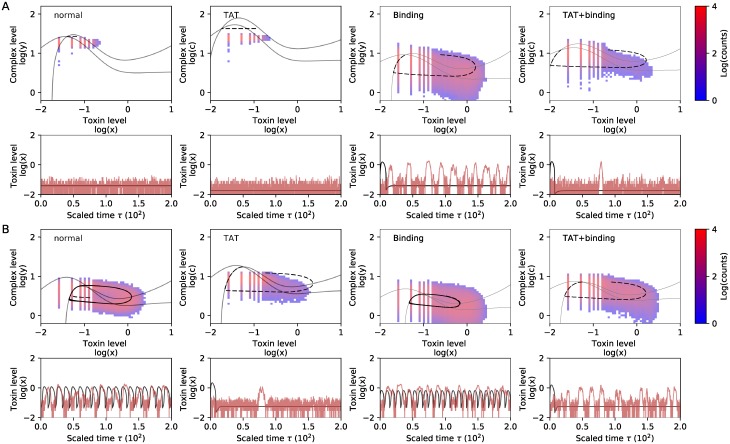
Inclusion of secondary complex formation and trancription regulation by DNA binding does not change the qualitative dynamics. In the simulations with DNA binding we used *r* = 4.9 as binding parameter, see S1 File. A: Simulations for the different models using *δ*_*a*_ = 1.5. The normal model and the model with complex TAT are not excitable here, while inclusion of DNA binding makes the system excitable for this stress level. B: Simulations for the different models using *δ*_*a*_ = 3. Here the normal model is excitable. Inclusion of complex TAT results in a lower excitation frequency as this has a stabilizing effect. This is observed as well for the model with DNA binding and complex TAT.

The only difference between these equations and (3) are the terms of *α*(*a*+ *y*)*x*. However, during an excitation the terms of order O(*ε*) become important, which have the same shape as in (3). Hence, the mechanism of the excitation does not change by inclusion of the secondary complex *z*. This is explored graphically in Fig 5, where we show the phase plane representation and the corresponding time traces for the deterministic and stochastic models, for low (*δ*_*a*_ = 1.5) and high (*δ*_*a*_ = 3) stress levels. The altered term −*α*(*a* + *y*)*x* does have a stabilizing effect on the system dynamics, as *x* will be depleted more quickly. This causes a shift in the nullclines such that the excitation threshold is larger if the secondary complex is included in the model (Fig 5A). Therefore, the excitation frequency is lower than in the reduced model when stress is increased (Fig 5B). The plotted nullclines in Fig 5 are approximations, assuming that *a* and *y* are of the same order of magnitude. This way the terms *α*(*a* + *y*)*x* reduce to 2*αax*. As a result there is not an exact correspondence with the intersection of the nullclines and the fixed point of the system.

#### Inclusion of binding to DNA

Transcriptional regulation occurs when the complex AT (*y*) binds to the DNA and thereby inhibits the transcription of mRNA. As such, the translation of the toxins and antitoxins is also indirectly inhibited. In the deterministic system, we model DNA binding by incorporating an inhibiting Hill function *r*/(*r* + *y*) into the translation rate (for derivation, see supporting information, S1 File). This expression decreases from 1 (no suppression) to 0 (complete suppression) for increasing values of the complex *y*. When assuming quasi steady state conditions for *m* and *a*, the system is still described by (3), but the expression for *m*(*x*) is changed as follows:

$$\begin{array}{c} m\left(x\right)=\frac{1}{\varepsilon }\frac{r}{r+y}\frac{1}{\left(1+\left(\beta -1\right)\frac{x^{n}}{x^{n}+\kappa^{n}}\right)} \end{array}$$ (9)

As the binding inhibits the translation of toxin and antitoxin, there is less complex formation. This causes a shift in the nullclines leading to an equilibrium with less complexes (Fig 5). Binding of AT to the DNA did not stabilize the system as the threshold for toxin excitations did not increase. Moreover, it seems that inclusion of DNA binding leads to excitations for parameter values where the reduced system is still stable (Fig 5A). Even though there is no stabillizing effect, the advantage of DNA binding is that the cell uses less energy as there is less translation [15]. If both the secondary complex TAT and DNA binding are included in the system, there are less complexes due to the binding and the system is more stable due to the presence of the secondary complex TAT (Fig 5A). When the amount of stress is increased, the excitation frequency is increased as well (Fig 5B), as is the case in the reduced model.

### Inclusion of growth rate inhibition allows for excitability without a sigmoidal response of mRNA cleavage by toxins

For the simulations we kept the Hill-coefficient fixed at *n* = 2. A Hill-coefficient larger than one leads to the bend in the nullclines, so that an unstable branch of NC(x) exists in the phase plane (see also S1 Fig). As explained before, this determines a threshold for an excitation.

When the Hill-coefficient is equal to one, the toxin dependence of the mRNA cleavage is hyperbolic rather than sigmoidal. As a result, the shape of the nullclines is such that no excitable behavior is observed in the models used above. However, even in this case, the system can still be excitable if we include extra biologically relevant mechanisms in these models.

**Fig 6:**
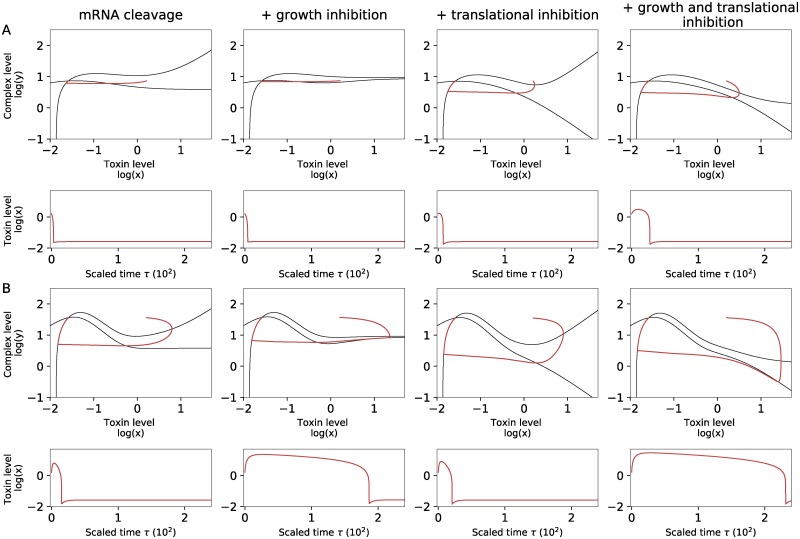
Influence of growth rate slowing down and translational inhibition for different values of the Hill coefficient. A: Simulations for the different models using *n* = 1. Without additional inhibition functions there is no excitable behavior. Translational inhibition in combination with mRNA cleavage can cause excitations. The excitation time is longer when growth rate inhibition is included. B: Simulations for the different models using *n* = 2. Excitable behavior is possible in all cases, with and without translational inhibition. Growth rate inhibition causes the cell to remain longer in the toxic state. Parameters: *B*_*t*_ = 1, *B*_*m*_ = 1, *S*_*t*_ = 1, *S*_*m*_ = 1.

One such mechanism is the slowing down of the growth rate due to increased toxin levels. Cataudella et al. have shown that growth rate inhibition, in combination with conditional cooperativity potentially leads to bistable behavior [13]. We used similar functions as in [13] to incorporate these effects in our model: the translation rate is scaled with a function $f_{m}=\frac{S_{m}}{1+B_{m}x}$, whereas the dilution rate is scaled with a function $f_{t}=\frac{S_{t}}{1+B_{t}x}$ (for the detailed model, see supporting information S1 File). In Fig 6 we study the effect of these inhibitory functions for Hill coefficient *n* = 1 (Fig 6A) and for Hill coefficient *n* = 2 (Fig 6B). In contrast to the result of Cataudella et al. [13], we do not find bistability when including these inhibiton functions, which is probably related to the fact that we use different time scales in our model than Cataudella et al., where it is assumed that complex formation happens instantaneously. The growth rate inhibition leads to increased excitation times, corresponding to the observation in Fig 1F. We find that for *n* = 1 translational inhibition is needed for excitable behavior. The decrease of the dilution rate does not affect the presence of excitable behavior, but causes the cell to be in the toxic regime for a longer period of time. In all the cases where excitable behavior exists, the nonlinearity of the nullclines is of at least degree two, causing the characteristic bend in the nullcline NC(x). This can be obtained by combining mRNA cleavage with *n* = 1 and translational inhibition, or by using mRNA cleavage with a Hill-coefficient of at least two.

## Conclusions

TA modules are small dynamic systems, coding for a toxin and its corresponding antitoxin [1–3]. The toxin level can affect a cell in different ways: post-segregational killing, abortive phage infections and the formation of persister cells [3, 10], although the latter is currently unclear [3, 12]. There exist different dynamic explanations for elevated toxin levels in cells. The first possibility is that this corresponds to a second equilibrium state, due to bistable behavior [13, 14, 16, 17], while the second possibility is that stochastic perturbations cause pulses in the free toxin level, corresponding to excitable behavior [15]. Recently it was found that mRNA cleavage by the toxins can cause toxin excitations, leading to cell growth heterogeneity [19].

In this article we used a deterministic model to analyze the role of mRNA cleavage as a possible mechanism behind such excitations. As there is a difference in time scales between toxin and antitoxin translation and degradation, the model can be simplified to a set of two ordinary differential equations (ODEs), allowing a description in the phase plane. An excitation occurs when a threshold is crossed, mRNA cleavage is switched on, and the repression of translation prevents an immediate return to the equilibrium state. By systematically comparing with Gillespie simulations we showed that even though the real system is inherently stochastic, a deterministic model is capable to describe the observed dynamics. When molecule numbers are low, a deterministic limit of a stochastic model does not always give an accurate description of the dynamics in the presence of multiple time scales [18, 39, 40]. Moreover, it has been shown that oscillatory behavior in noisy systems can be created by the noise, when no oscillations are observed in the deterministic model [33–35]. In this article, we show that the toxin excitations are caused by the underlying deterministic excitability, due to the vicinity of a Hopf bifurcation where a limit cycle is created. Stress can be modeled by varying the antitoxin degradation rate and the toxin translation rate, which increases the probability of excitations, as the fixed point gets closer to the unstable branch in the phase plane. In conclusion, even though this system is inherently stochastic, we provided a deterministic description of the excitable behavior in TA modules due the presence of toxin-induced cleavage of mRNA.

Similar excitable behavior in bacteria was theoretically and experimentally analyzed in the ComK—ComS gene regulatory circuit in *Bacillus subtilis*, where excitability led cells to be in a transient state in which they were competent to take up DNA from the environment [41, 42]. Although the circuitry of interacting genes and proteins in the ComK—ComS system is significantly different than that of the TA systems we studied here, the type of excitable behavior is similarly caused by a combination of fast positive and slow negative feedback loops. By using quantitative fluorescence time-lapse microscopy to observe circuit components in individual cells, and comparing such measurements with mathematical models, significant new insights were gained into how the ComK—ComS gene regulatory circuit works [41, 42]. Excitations in the free toxin level due to a difference in time scales also occur in the context of type I toxin-antitoxin modules, and they play a role in plasmid maintenance due to post-segregational killing [43, 44].

We hope that our model will trigger new experimental efforts in the field of TA systems, especially to measure the dynamics of circuit components on a single cell level, and that they will help in shedding new light on the temporal dynamics of cellular toxin and antitoxin levels and growth rates. Devising the necessary fluorescent reporters to track these protein levels *in vivo* is however no trivial task [45]. The reason is that adding a fluorescent tag to the toxin or the antitoxin may significantly alter the working of the operon, since both are small proteins, which have to be able to interact with each other, the DNA (for the antitoxin) and the target (for the toxin). An alternative approach is the use of a transcriptional fluorescent reporter, in which the promoter of the toxin-antitoxin system is fused to a fluorescent reporter gene, giving information about the transcription from the operon instead of the protein levels. However, many stress conditions interfere with translation, which may lower the readout from this type of fluorescent reporter. Still, we believe that such efforts would be very valuable, since they would allow to bridge the internal dynamics in individual cells and the dynamics on the level of whole cell populations, where bimodal distributions of fast and slowly dividing cells have been observed [19].

## Supporting information

S1 Fig Influence of the parameter *n*, *β*, *κ* and *α* on the phase plane. These parameters can change the shapes of the nullclines: an increase in *n* makes the slope steaper, an increase of *β* leads to a bigger difference between the nullclines in the toxic and normal state, *κ* changes the threshold itself and *α* affects the complex level at steady state. However, the overall behavior as explained in the main text remains the same.
(EPS)

S2 Fig Influence of an increase of *ε* for *δ*_*a*_ = 2. Comparison between the deterministic and the Gillespie simulations. An increase in stress by varying *ε* leads subsequently to excitability and oscilatory behavior. The Gillespie simulations do not always reach the fixed point when this is situated near the top of the nullclines (*ε* = 0.025, *ε* = 0.05 and *ε* = 0.075), as an excitation will occur as soon as the unstable branch is crossed. The data converges to the fixed point more closely when this is not situated in the top of the nullclines (e.g. *ε* = 0.15).
(EPS)

S1 File Model descriptions. A description of the different deterministic and corresponding Gillespie models that are used throughout the main text.
(PDF)

S2 File Analytic results of the TA system. Description how to find an analytic solution for the excitation and a discussion about the behavior of the system for *x* ≪ *κ*.
(PDF)

## References

[1] Pandey DP, Gerdes K. Toxin-antitoxin loci are highly abundant in free-living but lost from host-associated prokaryotes. Nucleic Acids Research. 2005;33(3):966–976. 10.1093/nar/gki201 15718296 PMC549392

[2] Page R, Peti W. Toxin-antitoxin systems in bacterial growth arrest and persistence. Nature Chemical Biology. 2016;12(4):208–214. 10.1038/nchembio.2044 26991085

[3] Harms A, Brodersen DE, Mitarai N, Gerdes K. Toxins, Targets, and Triggers: An Overview of Toxin-Antitoxin Biology. Molecular Cell. 2018;70(5):768–784. 10.1016/j.molcel.2018.01.003 29398446

[4] Gerdes K, Maisonneuve E. Bacterial persistence and toxin-antitoxin loci. Annual Review of Microbiology. 2012;66:103–123. 10.1146/annurev-micro-092611-150159 22994490

[5] Leplae R, Geeraerts D, Hallez R, Guglielmini J, Drèze P, Van Melderen L. Diversity of bacterial type II toxin-antitoxin systems: a comprehensive search and functional analysis of novel families. Nucleic Acids Research. 2011;39(13):5513–5525. 10.1093/nar/gkr131 21422074 PMC3141249

[6] Bernard P, Couturier M. Cell killing by the F plasmid CcdB protein involves poisoning of DNA-topoisomerase II complexes. Journal of Molecular Biology. 1992;226(3):735–745. 10.1016/0022-2836(92)90629-X 1324324

[7] Christensen SK, Pedersen K, Hansen FG, Gerdes K. Toxin-antitoxin loci as stress-response-elements: ChpAK/MazF and ChpBK cleave translated RNAs and are counteracted by tmRNA. Journal of Molecular Biology. 2003;332(4):809–819. 10.1016/S0022-2836(03)00922-7 12972253

[8] Zhang X, Yang Z, Khan SI, Horton JR, Tamaru H, Selker EU, et al Structural basis for the product specificity of histone lysine methyltransferases. Molecular Cell. 2003;12(1):177–185. 10.1016/S1097-2765(03)00224-7 12887903 PMC2713655

[9] Pedersen K, Zavialov AV, Pavlov MY, Elf J, Gerdes K, Ehrenberg M. The Bacterial Toxin RelE Displays Codon-Specific Cleavage of mRNAs in the Ribosomal A Site. Cell. 2003;112(1):131–140. 10.1016/S0092-8674(02)01248-5 12526800

[10] Van Melderen L. Toxin-antitoxin systems: why so many, what for? Current Opinion in Microbiology. 2010;13(6):781–785. 10.1016/j.mib.2010.10.006 21041110

[11] Lewis K. Persister cells. Annual Review of Microbiology. 2010;64:357–372. 10.1146/annurev.micro.112408.134306 20528688

[12] Goormaghtigh F, Fraikin N, Putrinš M, Hallaert T, Hauryliuk V, Garcia-Pino A, et al Reassessing the Role of Type II Toxin-Antitoxin Systems in Formation of Escherichia coli Type II Persister Cells. mBio. 2018;9(3):e00640–18. 10.1128/mBio.00640-18 29895634 PMC6016239

[13] Cataudella I, Sneppen K, Gerdes K, Mitarai N. Conditional Cooperativity of Toxin—Antitoxin Regulation Can Mediate Bistability between Growth and Dormancy. PLOS Computational Biology. 2013;9(8):e1003174 10.1371/journal.pcbi.1003174 24009488 PMC3757081

[14] Lou C, Li Z, Ouyang Q. A molecular model for persister in E. coli. Journal of Theoretical Biology. 2008;255(2):205–209. 10.1016/j.jtbi.2008.07.035 18721814

[15] Gelens L, Hill L, Vandervelde A, Danckaert J, Loris R. A General Model for Toxin-Antitoxin Module Dynamics Can Explain Persister Cell Formation in E. coli. PLOS Computational Biology. 2013;9(8):e1003190 10.1371/journal.pcbi.1003190 24009490 PMC3757116

[16] Feng J, Kessler DA, Ben-Jacob E, Levine H. Growth feedback as a basis for persister bistability. Proceedings of the National Academy of Sciences of the United States of America. 2014;111(1):544–549. 10.1073/pnas.1320396110 24344277 PMC3890803

[17] Tian C, Semsey S, Mitarai N. Synchronized switching of multiple toxin–antitoxin modules by (p)ppGpp fluctuation. Nucleic Acids Research. 2017;45(14):8180–8189. 10.1093/nar/gkx552 28854732 PMC5737467

[18] Thomas P, Straube AV, Grima R. Communication: Limitations of the stochastic quasi-steady-state approximation in open biochemical reaction networks. The Journal of Chemical Physics. 2011;135(18):181103 10.1063/1.3661156 22088045

[19] Nikolic N, Bergmiller T, Vandervelde A, Albanese TG, Gelens L, Moll I. Autoregulation of mazEF expression underlies growth heterogeneity in bacterial populations. Nucleic Acids Research. 2018;46(6):2918–2931. 10.1093/nar/gky079 29432616 PMC5888573

[20] Jones E, Oliphant T, Peterson P, et al. SciPy: Open source scientific tools for Python; 2001–. Available from: http://www.scipy.org/.

[21] Ben-Israel A. A Newton-Raphson method for the solution of systems of equations. Journal of Mathematical Analysis and Applications. 1966;15:243–252. 10.1016/0022-247X(66)90115-6

[22] Gillespie D. Exact stochastic simulation of coupled chemical-reactions. Journal of Physical Chemistry. 1977;81:2340–2361. 10.1021/j100540a008

[23] Kubitschek HE. Cell volume increase in Escherichia coli after shifts to richer media. Journal of Bacteriology. 1990;172(1):94–101. 10.1128/jb.172.1.94-101.1990 2403552 PMC208405

[24] Kamada K, Hanaoka F, Burley SK. Crystal structure of the MazE/MazF complex: molecular bases of antidote-toxin recognition. Molecular Cell. 2003;11(4):875–884. 10.1016/S1097-2765(03)00097-2 12718874

[25] Zorzini V, Buts L, Schrank E, Sterckx YGJ, Respondek M, Engelberg-Kulka H, et al Escherichia coli antitoxin MazE as transcription factor: insights into MazE-DNA binding. Nucleic Acids Research. 2015;43(2):1241–1256. 10.1093/nar/gku1352 25564525 PMC4333400

[26] Culviner PH, Laub MT. Global Analysis of the E. coli Toxin MazF Reveals Widespread Cleavage of mRNA and the Inhibition of rRNA Maturation and Ribosome Biogenesis. Molecular Cell. 2018;70(5):868–880.e10. 10.1016/j.molcel.2018.04.026 29861158 PMC8317213

[27] Santillán M, Mackey MC. Dynamic regulation of the tryptophan operon: A modeling study and comparison with experimental data. Proceedings of the National Academy of Sciences. 2001;98(4):1364–1369. 10.1073/pnas.98.4.1364 PMC29262 11171956

[28] Li GW, Burkhardt D, Gross C, Weissman JS. Quantifying Absolute Protein Synthesis Rates Reveals Principles Underlying Allocation of Cellular Resources. Cell. 2014;157(3):624–635. 10.1016/j.cell.2014.02.033 24766808 PMC4006352

[29] Schlosshauer M, Baker D. Realistic protein-protein association rates from a simple diffusional model neglecting long-range interactions, free energy barriers, and landscape ruggedness. Protein Science: A Publication of the Protein Society. 2004;13(6):1660–1669. 10.1110/ps.03517304 15133165 PMC2279981

[30] Schreiber G, Haran G, Zhou HX. Fundamental Aspects of Protein–Protein Association Kinetics. Chemical Reviews. 2009;109(3):839–860. 10.1021/cr800373w 19196002 PMC2880639

[31] Bernstein JA, Khodursky AB, Lin PH, Lin-Chao S, Cohen SN. Global analysis of mRNA decay and abundance in Escherichia coli at single-gene resolution using two-color fluorescent DNA microarrays. Proceedings of the National Academy of Sciences. 2002;99(15):9697–9702. 10.1073/pnas.112318199 PMC124983 12119387

[32] Lindner B, García-Ojalvo J, Neiman A, Schimansky-Geier L. Effects of noise in excitable systems. Physics Reports. 2004;392:321–424. 10.1016/j.physrep.2003.10.015

[33] McKane AJ, Nagy JD, Newman TJ, Stefanini MO. Amplified Biochemical Oscillations in Cellular Systems. Journal of Statistical Physics. 2007;128(1-2):165–191. 10.1007/s10955-006-9221-9

[34] Guisoni N, Monteoliva D, Diambra L. Promoters Architecture-Based Mechanism for Noise-Induced Oscillations in a Single-Gene Circuit. PLOS ONE. 2016;11(3):e0151086 10.1371/journal.pone.0151086 26958852 PMC4784906

[35] Thomas P, Straube AV, Timmer J, Fleck C, Grima R. Signatures of nonlinearity in single cell noise-induced oscillations. Journal of Theoretical Biology. 2013;335:222–234. 10.1016/j.jtbi.2013.06.021 23831270

[36] Christensen SK, Mikkelsen M, Pedersen K, Gerdes K. RelE, a global inhibitor of translation, is activated during nutritional stress. Proceedings of the National Academy of Sciences. 2001;98(25):14328–14333. 10.1073/pnas.251327898 PMC64681 11717402

[37] Dumortier F, Roussarie R. Canard cycles and center manifolds. vol. 121 of Memoirs of the American Mathematical Society. American Mathematical Society; 1996 Available from: https://www.ams.org/home/page/.

[38] Rué P, Garcia-Ojalvo J. Gene circuit designs for noisy excitable dynamics. Mathematical Biosciences. 2011;231(1):90–97. 10.1016/j.mbs.2011.02.013 21419138

[39] Thomas P, Straube AV, Grima R. The slow-scale linear noise approximation: an accurate, reduced stochastic description of biochemical networks under timescale separation conditions. BMC Systems Biology. 2012;6(1):39 10.1186/1752-0509-6-39 22583770 PMC3532178

[40] Berglund N, Gentz B. Noise-Induced Phenomena in Slow-Fast Dynamical Systems: A Sample-Paths Approach Probability and Its Applications. London: Springer-Verlag; 2006 Available from: https://www.springer.com/gp/book/9781846280382.

[41] Süel GM, Jordi GO, Liberman LM, Elowitz MB. An excitable gene regulatory circuit induces transient cellular differentiation. Nature. 2006;440(7083):545–550. 10.1038/nature04588 16554821

[42] Süel GM, Kulkarni RP, Dworkin J, Garcia-Ojalvo J, Elowitz MB. Tunability and noise dependence in differentiation dynamics. Science (New York, NY). 2007;315(5819):1716–1719. 10.1126/science.1137455 17379809

[43] Gong CC, Klumpp S. Modeling sRNA-Regulated Plasmid Maintenance. PLOS ONE. 2017;12(1):e0169703 10.1371/journal.pone.0169703 28085919 PMC5234814

[44] Himeoka Y, Mitarai N. Modeling slow-processing of toxin messenger RNAs in type-I toxin-antitoxin systems: post-segregational killing and noise filtering. Physical Biology. 2019;16(2):026001 10.1088/1478-3975/aaf3e3 30523873

[45] Nikolic N. Autoregulation of bacterial gene expression: lessons from the MazEF toxin–antitoxin system. Current Genetics. 2018 10.1007/s00294-018-0879-8 30132188 PMC6343021

