# Supplementary material for: Excitable dynamics through toxin-induced mRNA cleavage in bacteria
Source: PLoS One. 2019 Feb 22;14(2):e0212288. doi: 10.1371/journal.pone.0212288 (PMC6386449; doi:10.1371/journal.pone.0212288)
Supplement: S1 File — A description of the different deterministic and corresponding Gillespie models that are used throughout the main text. (PDF) [file pone.0212288.s003.pdf]

# Toxin excitations through toxin-induced mRNA cleavage
# Supplementary Information 1: Model descriptions

Stefan Vet  Alexandra Vandervelde  Lendert Gelens

January 3, 2019

## Deterministic model

### Model without TAT and without binding of AT to DNA

As explained in the main text, the differential equations with normalized variables are the following:

$$
\begin{aligned}
\frac{dm}{d\tau} &= \left(\frac{1}{\varepsilon} - m - (\beta - 1)\frac{x^n}{x^n + \kappa^n}m\right),\\
\frac{da}{d\tau} &= -\alpha a x + \gamma m - \delta_a a,\\
\frac{dx}{d\tau} &= -\alpha a x + \varepsilon\left(\gamma m + \delta_{AT} y - \delta_c x\right),\\
\frac{dy}{d\tau} &= \alpha a x - \varepsilon(\delta_c + \delta_{AT})y.
\end{aligned}
\tag{1}
$$

### Inclusion of TAT

TAT can be introduced by adding an additional normalized variable $z = \frac{d_m}{\varepsilon}[TAT]$ to the differential equations in the following way:

$$\begin{aligned}
m(x) &= \frac{1}{\varepsilon\left(1+(\beta-1)\frac{x^n}{x^n+\kappa^n}\right)}, \\
a(x) &= \frac{\gamma m(x)}{\alpha x+\delta_a}, \\
\frac{dx}{d\tau} &= -\alpha(a(x)+y)x+\varepsilon\left(\gamma m(x)+\delta_{AT}(y+2z)-\delta_c x\right), \\
\frac{dy}{d\tau} &= \alpha(a(x)-y)x-\varepsilon(\delta_c+\delta_{AT})y, \\
\frac{dz}{d\tau} &= \alpha xy-\varepsilon(\delta_c+\delta_{AT})z.
\end{aligned} \tag{2}$$

As explained in the main text $z$ is created when $x$ and $y$ bind, where we assume for simplicity that this complex is created and degraded with the same rates ($\alpha$, $\delta_c$ and $\delta_{AT}$) as $y$. We define the total amount of toxin in the complexes, $c = y + 2z$, resulting in the following system:

$$\begin{aligned}
m(x) &= \frac{1}{\varepsilon\left(1+(\beta-1)\frac{x^n}{x^n+\kappa^n}\right)}, \\
a(x) &= \frac{\gamma m(x)}{\alpha x+\delta_a}, \\
\frac{dx}{d\tau} &= -\alpha(a(x)+y)x+\varepsilon\left(\gamma m(x)+\delta_{AT}c-\delta_c x\right), \\
\frac{dc}{d\tau} &= \alpha(a(x)+y)x-\varepsilon(\delta_c+\delta_{AT})c.
\end{aligned} \tag{3}$$

## Modeling binding of AT to DNA

To model DNA binding, we introduce additional equations to the original system. These describe the unbound DNA $[D]$ and the bound DNA $[D_{AT}]$.

$$\begin{aligned}
\frac{d[D]}{dt} &= c_2[D_{AT}]-c_1[AT][D], \\
\frac{d[D_{AT}]}{dt} &= c_1[AT][D]-c_2[D_{AT},] \\
\frac{d[M]}{dt} &= r_F[D]-d_m[M]-(\beta-1)d_m\frac{[T]^n}{[T]^n+K_t^n}[M], \\
\frac{d[A]}{dt} &= b_1[M]-a_T[A][T]-d_a[A], \\
\frac{d[T]}{dt} &= \varepsilon b_1[M]-a_T[A][T]-d_c[T]+d_{a2}[AT], \\
\frac{d[AT]}{dt} &= a_T[A][T]-d_c[AT]-d_{a2}[AT].
\end{aligned} \tag{4}$$

Hence DNA binds with AT with a rate $c_1 = 1.28\cdot 10^{-05}s^{-1}$, while the unbinding occurs with a rate $c_2 = 2.31\cdot 10^{-03}s^{-1}$.

Assuming $D$ and $D_{AT}$ are in equilibrium, then

$$\begin{aligned}\frac{d[D]}{dt} &= c_2[D_{AT}] - c_1[AT][D] = 0,\\ \frac{d[D_{AT}]}{dt} &= c_1[AT][D] - c_2[D_{AT}] = 0.\end{aligned} \tag{5}$$

The DNA is either bound or unbound, so that $[D] + [D_{AT}] = 1$. Using this information, then

$$[D] = \frac{c_2}{c_2 + c_1[AT]} \tag{6}$$

Therefore:

$$\begin{aligned}\frac{d[M]}{dt} &= r_F \frac{c_2}{c_2 + c_1[AT]} - d_m[M] - (\beta - 1)d_m \frac{[T]^n}{[T]^n + K_t^n}[M],\\ \frac{d[A]}{dt} &= b_1[M] - a_T[A][T] - d_a[A],\\ \frac{d[T]}{dt} &= \varepsilon b_1[M] - a_T[A][T] - d_c[T] + d_{a2}[AT],\\ \frac{d[AT]}{dt} &= a_T[A][T] - d_c[AT] - d_{a2}[AT].\end{aligned} \tag{7}$$

After normalization we obtain the following equations:

$$\begin{aligned}\frac{dm}{d\tau} &= \left(\frac{1}{\varepsilon}\frac{r}{r+y} - m - (\beta - 1)\frac{x^n}{x^n + \kappa^n}m\right),\\ \frac{da}{d\tau} &= -\alpha a x + \gamma m - \delta_a a,\\ \frac{dx}{d\tau} &= -\alpha a x + \varepsilon\left(\gamma m + \delta_{AT} y - \delta_c x\right),\\ \frac{dy}{d\tau} &= \alpha a x - \varepsilon(\delta_c + \delta_{AT})y.\end{aligned} \tag{8}$$

Where $r = \frac{d_m c_2}{\varepsilon c_1} = 4.9$.

Assuming $m$ and $a$ are in equilibrium, this becomes:

$$\begin{aligned}m(x) &= \frac{1}{\varepsilon}\frac{r}{r+y}\frac{1}{\left(1 + (\beta - 1)\frac{x^n}{x^n + \kappa^n}\right)},\\ a(x) &= \frac{\gamma m(x)}{\alpha x + \delta_a},\\ \frac{dx}{d\tau} &= -\alpha a(x) x + \varepsilon\left(\gamma m(x) + \delta_{AT} y - \delta_c x\right),\\ \frac{dy}{d\tau} &= \alpha a(x) x - \varepsilon(\delta_c + \delta_{AT})y.\end{aligned} \tag{9}$$

## Modeling binding of AT to DNA, with TAT included.

Combining the results of the two former sections, we obtain:

$$
\begin{aligned}
m(x) &= \frac{r}{r+y}\frac{1}{\varepsilon}\frac{1}{\left(1+(\beta-1)\frac{x^n}{x^n+\kappa^n}\right)},\\
a(x) &= \frac{\gamma m(x)}{\alpha x+\delta_a},\\
\frac{dx}{d\tau} &= -\alpha(a(x)+y)x+\varepsilon\left(\gamma m(x)+\delta_{AT}c-\delta_c x\right),\\
\frac{dc}{d\tau} &= \alpha(a(x)+y)x-\varepsilon(\delta_c+\delta_{AT})c.
\end{aligned} \tag{10}
$$

## Inclusion of growth rate slowing down and translational inhibition

We introduce the inhibition on the translation of mRNA ($f_m$) and the inhibition of the growth ($f_t$) by using the following functions:

$$
\begin{aligned}
f_m &= \frac{S_m}{1+B_m x}\\
f_t &= \frac{S_t}{1+B_t x}
\end{aligned} \tag{11}
$$

These functions are based on Cataudella et al.,PLoS Computational Biology, 2013. Leaving out secondary complex formation and DNA binding, the set of differential equations then becomes:

$$
\begin{aligned}
\frac{dm}{d\tau} &= \left(\frac{1}{\varepsilon}-m-(\beta-1)\frac{x^n}{x^n+\kappa^n}m\right),\\
\frac{da}{d\tau} &= -\alpha ax+\gamma m f_m(x)-\delta_a a,\\
\frac{dx}{d\tau} &= -\alpha ax+\varepsilon\left(\gamma m f_m(x)+\delta_{AT}y-\delta_c x f_t(x)\right),\\
\frac{dy}{d\tau} &= \alpha ax-\varepsilon(\delta_c f_t(x)+\delta_{AT})y.
\end{aligned} \tag{12}
$$

In two dimensions this is:

$$
\begin{aligned}
m &= \frac{1}{\varepsilon\left(1-(\beta-1)\frac{x^n}{x^n+\kappa^n}\right)}, \\
a &= \frac{\gamma m}{\alpha x+\delta_a} f_m(x), \\
\frac{dx}{d\tau} &= -\alpha a x+\varepsilon\left(\gamma m f_m(x)+\delta_{AT} y-\delta_c x f_t(x)\right), \\
\frac{dy}{d\tau} &= \alpha a x-\varepsilon(\delta_c f_t(x)+\delta_{AT}) y.
\end{aligned} \tag{13}
$$

# Stochastic model

## Model without TAT and without binding of AT to DNA

Table 1: **Core reactions for the Gillespie simulations.**

| Protein Level Reaction | Propensity | $M$ | $A$ | $T$ | $AT$ | $TAT$ | $D$ | $D_{AT}$ |
|---|---|---|---|---|---|---|---|---|
| mRNA Creation | $r_F D$ | +1 | 0 | 0 | 0 | 0 | 0 | 0 |
| Antitoxin translation | $b_1 M$ | 0 | +1 | 0 | 0 | 0 | 0 | 0 |
| Toxin translation | $b_2 M$ | 0 | 0 | +1 | 0 | 0 | 0 | 0 |
| Decay mRNA | $(d_m + d_{large} * \frac{T^{n_H}}{T^{n_H}+K_T^{n_H}})M$ | -1 | 0 | 0 | 0 | 0 | 0 | 0 |
| Decay antitoxin | $d_A A$ | 0 | -1 | 0 | 0 | 0 | 0 | 0 |
| Decay toxin | $d_C T$ | 0 | 0 | -1 | 0 | 0 | 0 | 0 |
| Decay complex AT | $d_C AT$ | 0 | 0 | 0 | -1 | 0 | 0 | 0 |
| Complex AT formation | $a_T A * T$ | 0 | -1 | -1 | +1 | 0 | 0 | 0 |
| Decay of A within complex AT | $d_{a2} AT$ | 0 | 0 | +1 | -1 | 0 | 0 | 0 |

In the absence of DNA binding, $D = 1$.

## Inclusion of TAT

Table 2: **Additional reactions for the secondary toxin-antitoxin complex TAT**

| Protein Level Reaction | Propensity | $M$ | $A$ | $T$ | $AT$ | $TAT$ | $D$ | $D_{AT}$ |
|---|---|---|---|---|---|---|---|---|
| Decay toxin-antitoxin complex TAT | $d_C TAT$ | 0 | 0 | 0 | 0 | -1 | 0 | 0 |
| Complex TAT formation | $a_T T * AT$ | 0 | 0 | -1 | -1 | +1 | 0 | 0 |
| Decay of A within complex TAT | $d_{a2} TAT$ | 0 | 0 | +2 | 0 | -1 | 0 | 0 |

## Modeling binding of AT to DNA

Table 3: **Additional reactions for the DNA binding.**

| Protein Level Reaction | Propensity | $M$ | $A$ | $T$ | $AT$ | $TAT$ | $D$ | $D_{AT}$ |
|---|---|---|---|---|---|---|---|---|
| Binding of AT to DNA | $c_1 * AT * D$ | 0 | 0 | 0 | 0 | -1 | -1 | +1 |
| Unbinding of AT from DNA | $c_2 * D_{AT}$ | 0 | 0 | 0 | 0 | +1 | +1 | -1 |

## Inclusion of the toxic effect on the growth rate and translational inhibition

The full model becomes:

Table 4: **Full reactions for the Gillespie simulations.**

| Protein Level Reaction | Propensity | $M$ | $A$ | $T$ | $AT$ | $TAT$ | $D$ | $D_{AT}$ |
|---|---|---|---|---|---|---|---|---|
| mRNA Creation | $r_F D$ | +1 | 0 | 0 | 0 | 0 | 0 | 0 |
| Antitoxin translation | $b_1 M \frac{s_m}{1+s_m T}$ | 0 | +1 | 0 | 0 | 0 | 0 | 0 |
| Toxin translation | $b_2 M \frac{s_m}{1+b_m T}$ | 0 | 0 | +1 | 0 | 0 | 0 | 0 |
| Decay mRNA | $(d_m + d_{large} * \frac{T^{n_H}}{T^{n_H}+K_T^{n_H}})M$ | -1 | 0 | 0 | 0 | 0 | 0 | 0 |
| Decay antitoxin | $d_A A$ | 0 | -1 | 0 | 0 | 0 | 0 | 0 |
| Decay toxin | $d_C T \frac{s_t}{1+s_t T}$ | 0 | 0 | -1 | 0 | 0 | 0 | 0 |
| Decay complex AT | $d_C AT \frac{s_t}{1+b_t T}$ | 0 | 0 | 0 | -1 | 0 | 0 | 0 |
| Complex AT formation | $a_T A * T$ | 0 | -1 | -1 | +1 | 0 | 0 | 0 |
| Decay of A within complex AT | $d_{a2} AT$ | 0 | 0 | +1 | -1 | 0 | 0 | 0 |
| Decay toxin-antitoxin complex TAT | $d_C TAT \frac{s_t}{1+b_t T}$ | 0 | 0 | 0 | 0 | -1 | 0 | 0 |
| Complex TAT formation | $a_T T * AT$ | 0 | 0 | -1 | -1 | +1 | 0 | 0 |
| Decay of A within complex TAT | $d_{a2} TAT$ | 0 | 0 | +2 | 0 | -1 | 0 | 0 |
| Binding of AT to DNA | $c_1 * AT * D$ | 0 | 0 | 0 | 0 | -1 | -1 | +1 |
| Unbinding of AT from DNA | $c_2 * D_{AT}$ | 0 | 0 | 0 | 0 | +1 | +1 | -1 |

Note: the parameters $s_t$, $s_m$, $b_t$ and $b_m$ are linked to the parameters that we used in Eq 11 in the following way: $s_t = S_t$, $s_m = S_m$, $b_t = \frac{d_m}{\varepsilon} B_t$ and $b_m = \frac{d_m}{\varepsilon} B_m$.

## Estimating the amplitude, period and excitation time for the stochastic simulations

First, we apply a smoothing to the timetrace for the normalized toxin concentration using the rloess method in Matlab. To estimate the duration of a toxin excitation, we take the average duration of the episodes when the smoothed normalized toxin concentration is higher than $\kappa$, the normalized threshold for the toxic effect on the mRNA. To estimate the amplitude, we take the average of the maxima of these episodes. To estimate the period, we take the average of the times between the start of each two episodes when the smoothed normalized toxin concentration is higher than $\kappa$.
