# Supplementary material for: Excitable dynamics through toxin-induced mRNA cleavage in bacteria
Source: PLoS One. 2019 Feb 22;14(2):e0212288. doi: 10.1371/journal.pone.0212288 (PMC6386449; doi:10.1371/journal.pone.0212288)
Supplement: S2 File — Description how to find an analytic solution for the excitation and a discussion about the behavior of the system for x ≪ κ. (PDF) [file pone.0212288.s004.pdf]

Toxin excitations through toxin-induced mRNA cleavage
Supplementary Information 2: Analytical estimates

Stefan Vet Alexandra Vandervelde Lendert Gelens

January 3, 2019

This supplementary text consists of three parts. First we will discuss an analytic estimate of the time evolution of a toxin excitation. In the second part we will show that there is a different time scale separation in the regime $x < \kappa$ than the one used in the main text. Finally we show that in this regime where $x < \kappa$ it is possible to estimate the fixed point and show that it is always stable for $x \ll \kappa$ by calculating its eigenvalues.

Analytic estimate of a toxin excitation

We start from the simplified system:

$$\begin{aligned}
m(x) &= \frac{1}{\varepsilon\left(1 + (\beta - 1)\frac{x^n}{x^n+\kappa^n}\right)},\\
a(x) &= \frac{\gamma m(x)}{\alpha x + \delta_a},\\
\frac{dx}{d\tau} &= -\alpha a(x)x + \varepsilon\left(\gamma m(x) + \delta_{AT} y - \delta_c x\right),\\
\frac{dy}{d\tau} &= \alpha a(x)x - \varepsilon(\delta_c + \delta_{AT})y.
\end{aligned} \tag{1}$$

During an excitation the toxin level is such that $x > \kappa$ and mRNA cleavage is switched on. In this situation, Eqs. (1) can be further simplified using following assumptions:

- Cleavage of mRNA (m) slows down the translation to a and x, so that $\gamma m \approx 0$.
- There is almost no creation of complexes (y) during an excitation due to the suppressed translation of antitoxins a, hence $\alpha a x \approx 0$.

These assumptions are not valid outside of the regime $x > \kappa$, so the following calculations are only valid to get a first approximation for the shape of the toxin excitation and do not describe the approach to the fixed point. Using these assumptions we can write simplified differential

equations for y and $x + y$:

$$\frac{dy}{d\tau} = -\varepsilon(\delta_c + \delta_{AT})y,$$
$$\frac{d(x+y)}{d\tau} = -\varepsilon\delta_c(x+y). \tag{2}$$

These are linear, univariable differential equations, of which the solutions are exponentially decaying functions, given by:

$$y(t) = y(0)e^{-\varepsilon(\delta_c+\delta_{AT})\tau},$$
$$x(t) + y(t) = (x(0) + y(0))e^{-\varepsilon\delta_c\tau}. \tag{3}$$

An equation for $x(t)$ is then found by substitution of y(t) in the last equation:

$$x(t) = (x(0) + y(0))e^{-\varepsilon\delta_c\tau} - y(0)e^{-\varepsilon(\delta_c+\delta_{AT})\tau}. \tag{4}$$

The toxin level x consists of two exponentially decaying functions with two different half times. The term $-y(0)e^{-\varepsilon(\delta_c+\delta_{AT})\tau}$, which initially supresses x, decreases more quickly than $(x(0) + y(0))e^{-\varepsilon\delta_c\tau}$, so that x increases initially. When the term $-y(0)e^{-\varepsilon(\delta_c+\delta_{AT})\tau}$ becomes small, x will go through a maximum and decrease exponentially according to $(x(0) + y(0))e^{-\varepsilon\delta_c\tau}$.

These equations allow to estimate the excitation time and amplitude length. The time when the excitation reaches a maximum, as well as the corresponding maximal amplitude, can be calculated by taking the time derivative of (4), which must be equal to zero. Solving for τ_{max} and $x(\tau_{max})$ yields:

$$\tau_{max} = -\frac{\ln\left(\frac{\delta_c}{\delta_c+\delta_{AT}}\frac{x(0)+y(0)}{y(0)}\right)}{\varepsilon\delta_{AT}},$$
$$x(\tau_{max}) = (x(0)+y(0))\left(\frac{\delta_c}{\delta_c+\delta_{AT}}\frac{x(0)+y(0)}{y(0)}\right)^{\frac{\delta_c}{\delta_{AT}}} - y(0)\left(\frac{\delta_c}{\delta_c+\delta_{AT}}\frac{x(0)+y(0)}{y(0)}\right)^{\frac{\delta_c+\delta_{AT}}{\delta_{AT}}}. \tag{5}$$

In Fig 1 the behavior of all variables are shown for the analytic result (A), the simplified system (1) (B), the four dimensional system (C) and the Gillespie simulations (D). There is a good correspondence of the simulated peak with the calculated time and amplitude (5).

Behavior in the regime $x < \kappa$: time scale separation

In the main text we used the following four dimensional system:

$$\frac{dm}{d\tau} = \left(\frac{1}{\varepsilon} - m - (\beta - 1)\frac{x^n}{x^n + \kappa^n}m\right)$$
$$\frac{da}{d\tau} = -\alpha ax + \gamma m - \delta_a a$$
$$\frac{dx}{d\tau} = -\alpha ax + \varepsilon\left(\gamma m + \delta_{AT}y - \delta_c x\right) \tag{6}$$
$$\frac{dy}{d\tau} = \alpha ax - \varepsilon(\delta_c + \delta_{AT})y$$

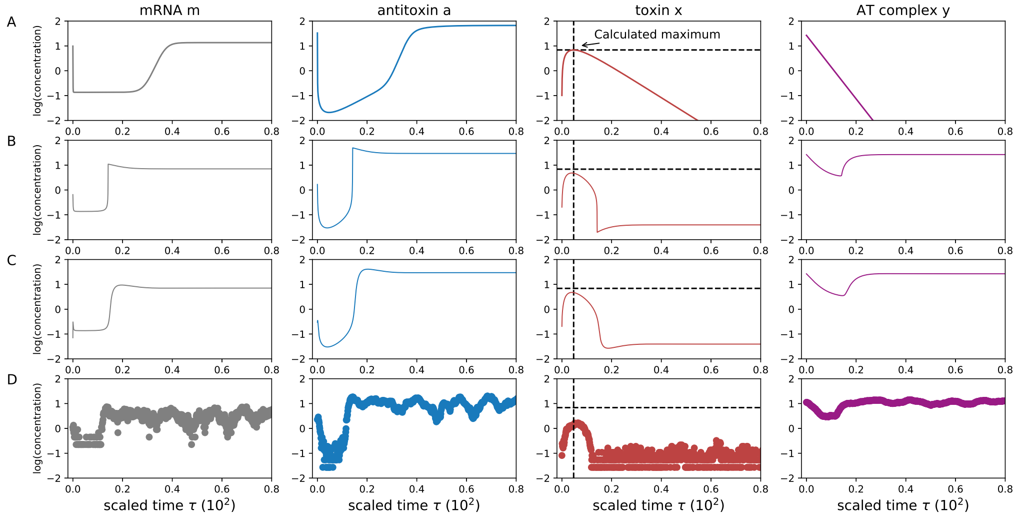

Figure 1: **Concentrations of mRNA (m), antitoxin (a), toxin (x) and the AT complex (y) as a function of time.** A. Analytic solution. The time and amplitude of the excursion of the toxin level x can be calculated using (5). B. Two dimensional ODE system C. Four dimensional ODE system D. Gillespie simulation. For x the maximum of the analytic excursion can

This was simplified to a two dimensional system, where quasi steady state conditions for m and a are assumed:

$$\begin{aligned}
m(x) &= \frac{1}{\varepsilon\left(1-(\beta-1)\frac{x^n}{x^n+\kappa^n}\right)} \\
a(x) &= \frac{\gamma m(x)}{\alpha x+\delta_a} \\
\frac{dx}{d\tau} &= -\alpha a(x)x+\varepsilon\left(\gamma m(x)+\delta_{AT}y-\delta_c x\right) \\
\frac{dy}{d\tau} &= \alpha a(x)x-\varepsilon(\delta_c+\delta_{AT})y
\end{aligned} \tag{7}$$

The difference between the two dimensional system (7) and the four dimensional system (6) can be seen in Fig 3 in the main text: in the regime where $x<\kappa$ the two dimensional system rapidly approaches the nullcline NC(x), whereas the approach in the four dimensional system is more smooth. In this section we will discuss this difference into more detail and show that the reason is that in this regime a different time scale separation applies. Due to the small values of x, the difference between both models is also very small, so that this does not largely influence the results obtained in the main text.

The system (6) was obtained by normalizing variables and parameters such that they are of order O(1), which is no longer the case for small values of x. Therefore x can be rescaled by defining a new variable $x'=\frac{x}{\varepsilon}$, such that the new differential equations become:

$$
\begin{aligned}
\frac{dm}{d\tau} &= \left(\frac{1}{\varepsilon} - m - (\beta - 1)\frac{x'^n}{x'^n + \kappa'^n} m\right) \\
\frac{da}{d\tau} &= -\varepsilon\alpha a x' + \gamma m - \delta_a a \\
\frac{dx'}{d\tau} &= -\alpha a x' + \gamma m + \delta_{AT} y - \varepsilon\delta_c x' \\
\frac{dy}{d\tau} &= \varepsilon(\alpha a x' - \delta_c + \delta_{AT}) y
\end{aligned}
\tag{8}
$$

Here $\kappa' = \frac{\kappa}{\varepsilon}$ and we see another difference in time scales than originally assumed. The toxin level x changes with terms of order O(1), whereas $\frac{dy}{d\tau}$ is of order O(ε). This difference can be observed in the two dimensional system, where x approaches the nullcline NC(x) very rapidly for $x < \kappa$. The approach is more smooth for the four dimensional system, where the quasi-steady state assumptions for m and a are not fulfilled. In the time course of Fig 3 (main text) it can be seen that this difference is very small, as it only exists for small values of x. Moreover, in the Gillespie data the relative variation on x in $x < \kappa$ will be large as there is a very small concentration of x. Therefore this difference does not influence the general behavior of the system.

Behavior in the regime $x < \kappa$, calculation of the fixed point and eigenvalues

Here we will estimate the fixed point in the regime $x \ll \kappa$ and show that it is always stable in this regime. Starting again from Eqs (7), the fixed point of the system are calculated by setting $\frac{dx}{d\tau} = \frac{dy}{d\tau} = 0$:

$$
\begin{aligned}
\frac{dx}{d\tau} &= -\alpha a x + \varepsilon(\gamma m + \delta_{AT} y - \delta_c x) = 0 \\
\frac{dy}{d\tau} &= \alpha a x - \varepsilon(\delta_c + \delta_{AT}) y = 0
\end{aligned}
\tag{9}
$$

These equations can be further simplified , as for $x \ll \kappa$: $m \approx \frac{1}{\varepsilon}$ and $a(x) \approx \frac{\gamma}{\varepsilon\delta_a}$. Moreover, the term $\delta_c x$ is neglible due to the small value of x. Hence:

$$
\begin{aligned}
\frac{dx}{d\tau} &= -\alpha a x + \gamma + \varepsilon\delta_{AT} y = 0 \\
\frac{dy}{d\tau} &= \alpha a x - \varepsilon(\delta_c + \delta_{AT}) y = 0
\end{aligned}
\tag{10}
$$

This becomes:

$$
\begin{aligned}
-\alpha\frac{\gamma}{\varepsilon\delta_a} x + \gamma + \varepsilon\delta_{AT} y = 0 \\
\alpha\frac{\gamma}{\varepsilon\delta_a} x - \varepsilon(\delta_c + \delta_{AT}) y = 0
\end{aligned}
\tag{11}
$$

Rewriting yields:

$$
\begin{aligned}
&-\alpha\gamma x+\varepsilon\delta_a\gamma+\varepsilon^2\delta_a\delta_{AT}y=0\\
&+\alpha\gamma x-\varepsilon^2(\delta_c+\delta_{AT})\delta_a y=0
\end{aligned}
\tag{12}
$$

Therefore the fixed point is estimated by following values of x and y:

$$
\begin{aligned}
x&=\varepsilon\frac{\delta_a}{\alpha}(1+\frac{\delta_{AT}}{\delta_c})\\
y&=\frac{\gamma}{\varepsilon\delta_c}
\end{aligned}
\tag{13}
$$

The jacobian of the system is then given by:

$$
J=\begin{bmatrix}-\alpha a & \varepsilon\delta_{AT}\\ \alpha a & -\varepsilon(\delta_c+\delta_{AT})\end{bmatrix}
\tag{14}
$$

So that the eigenvalues are given by the following functions:

$$
\lambda=\frac{-[\alpha a+\epsilon(\delta_c+\delta_{AT})]\pm\sqrt{(\alpha a+\varepsilon(\delta_c+\delta_{AT}))^2-4(\varepsilon\alpha a\delta_c)}}{2}<0
\tag{15}
$$

These are both negative, so that the fixed point is always stable, as long as it is in the regime that $x<\kappa$.
